# A Biomarker Panel for the Detection of Pancreatic Cancer

**DOI:** 10.3390/cancers18091397

**Published:** 2026-04-28

**Authors:** Yuefan Wang, Yuanyu Huang, Tung-Shing M. Lih, Christine Worthington, Zhenyu Sun, Lori J. Sokoll, Amer Zureikat, Alessandro Paniccia, Daniel W. Chan, Zhen Zhang, Randall E. Brand, Hui Zhang

**Affiliations:** 1 Department of Pathology, Johns Hopkins University School of Medicine, Baltimore, MD 21231, USA; 2 Department of Pathology, University of Pittsburgh Medical Center, Pittsburgh, PA 15261, USA; 3 Department of Surgery, University of Pittsburgh Medical Center, Pittsburgh, PA 15261, USA

**Keywords:** pancreatic cancer, blood proteomics, COF-enrichment, AUTO-SP, CA19-9, CEACAM1, ITIH3

## Abstract

**Background**: Pancreatic cancer is an aggressive malignancy with poor survival. Most patients are diagnosed at advanced or metastatic stages because early disease is often asymptomatic and effective screening tools are lacking. We evaluated a three-marker model comprising serum CA19-9 in combination with the plasma proteins ITIH3 and CEACAM1 for pancreatic ductal adenocarcinoma (PDAC) detection. **Methods**: Matched plasma and serum samples were collected from 649 participants (250 PDAC cases and 399 controls). Plasma proteins were enriched using high-surface area magnetic covalent organic framework (COF) polymers. Serum CA19-9 was measured using the Tosoh Bioscience immunoassay. The marker panel was trained using a radial-based SVM with repeated 10-fold cross-validation using a set-aside training sample set. The derived model along with a fixed cutoff corresponding to 95% sensitivity in training samples were independently validated using a blinded sample set. **Results**: In the independent blinded validation, the combined panel of serum CA19-9 with plasma ITIH3 and CEACAM1 achieved an AUC of 0.917 indicating that the three-marker panel maintained strong performance in distinguishing PDAC from controls. At the prefixed threshold, the three-marker panel had a specificity of 53.3% (95% CI: 46.8–59.7%), significantly outperforming CA19-9 alone at 14.5% (95% CI: 10.4–19.7%). **Conclusions**: In independently blinded validation, combining plasma ITIH3 and CEACAM1 with serum CA19-9 substantially improved diagnostic performance for PDAC, achieving high specificity while maintaining 95% sensitivity compared with serum CA19-9 alone. These findings support further validation of this three-marker panel as a potential PDAC monitoring and detection approach in larger, multicenter studies.

## Simple Summary

Detection of pancreatic cancer using a blood test is crucial because symptoms often appear at a late stage when cancer treatment options become limited. A blood test could help, since it is minimally invasive and can be repeated over time. Currently, the most widely used pancreatic cancer blood marker is serum CA19-9, but CA19-9 does not provide enough specificity for pancreatic cancer screening. To strengthen blood-based detection, we applied a magnetic polymer enrichment strategy that extends the measurable range of plasma proteins using proteomics to select promising candidates. In the current study, clinical performance of a panel of two plasma proteins, ITIH3 and CEACAM1, combined with serum CA19-9 in a multivariate model was evaluated on an independent blinded validation set. The model improved overall diagnostic specificity at 95% sensitivity over CA19-9 alone with statistical significance. If confirmed in larger multicenter studies, this panel either alone or in combination with other biomarkers could enable more specific detection of pancreatic cancer.

## 1. Introduction

Pancreatic cancer remains one of the most lethal malignancies [1,2], with persistently poor survival and a rising mortality burden compared with many other cancer types [3,4,5]. Early detection offers the opportunity to improve outcomes, as patients diagnosed with low-stage and localized disease have substantially better survival than those presenting with advanced or metastatic tumors [6,7,8]. However, the detection rate of early-stage pancreatic cancer is still low in routine clinical practice because it is often asymptomatic [9], and current diagnostic strategies are not sufficiently sensitive and specific for population-level screening or timely detection in at-risk individuals [10]. Pancreatic cancer has a low incidence in the general population [2,5], making population-wide screening impractical [8]. A more feasible strategy is to monitor and detect among high-risk individuals. However, the current standard biomarker assay for disease progression monitoring, CA19-9 [11,12,13,14], shows limited specificity when sensitivity is set high [15,16], so false positives are difficult to avoid under current conditions. These limitations underscore an urgent need for improved screening and detection approaches that can identify pancreatic cancer with greater precision, when curative intervention is more feasible and mortality can be reduced [17].

To improve detection of pancreatic cancer, we recently leveraged tissue-based proteomic and glycoproteomic profiling to identify pancreatic ductal adenocarcinoma (PDAC)-associated circulating glycoproteins in blood [18,19], as well as proteins and glycoproteins linked to pancreatic cancer progression [20], and multiplex immunoassays for pancreatic cancer detection [21,22]. In current clinical practice, carbohydrate antigen 19-9 (CA19-9) is measured using FDA-cleared immunoassays and is used as an aid in the management and monitoring of pancreatic cancer [17]. However, CA19-9 alone has limited specificity and can be elevated in several benign hepatobiliary and inflammatory conditions [12,23]. Therefore, we sought to integrate our proteomic-discovered biomarkers with CA19-9 to improve the diagnostic accuracy for the detection of pancreatic cancer.

Mass spectrometry (MS)-based blood proteomics has developed rapidly in recent years [24,25,26,27,28], and magnetic polymer enrichment strategies can substantially expand the detectable depth of plasma proteins [28,29,30]. Using our in-house synthetic magnetic covalent organic framework (COF) polymers [31,32,33], we enriched plasma proteins and achieved deep proteome coverage in our automated sample preparation (AUTO-SP) platform [34,35], enabling quantification of more than 5000 proteins in total. Building on this capability, we prioritized two plasma proteins, inter-alpha-trypsin inhibitor heavy chain H3 (ITIH3) and carcinoembryonic antigen-related cell adhesion molecule 1 (CEACAM1), which consistently emerged as robust PDAC-associated candidates in our prior studies [19,20,22], and evaluated whether incorporating them with CA19-9 could improve diagnostic performance. Because the previously published studies were conducted in an independent cohort collected at different sites, the reported associations of ITIH3 and CEACAM1 with PDAC in our cohort provide an external, cross-cohort validation at the individual biomarker level. Our results reflect the performance of these two markers in a distinct population and sample collection cohort, supporting their general applications.

We developed and evaluated a model consisting of a three-marker panel (CA19-9/ITIH3/CEACAM1) designed to increase specificity while maintaining high sensitivity for PDAC detection.

## 2. Materials and Methods

### 2.1. Plasma and Serum Collection, Sample Usage

Matched EDTA plasma and serum samples were collected from the same participants at the University of Pittsburgh Medical Center (UPMC) under the institutional review boards and in accordance with the Pancreatic Cancer Detection Consortium (PCDC)- approved standard operating procedures [36]. All samples used in this study were retrospectively obtained using established early detection research network (EDRN) biobanking protocols that banked all case specimens around PDAC diagnostic workup, temporally ranging from prior to histologic confirmation, after diagnostic imaging identifying the lesion to post histologic confirmation, at initial oncologist appointment; all case specimens were banked prior to initiation of any cancer-related therapy. Diagnoses and case classifications were determined using all clinical information available at the time of this study, and all PDAC participants had a histologically confirmed diagnosis. PDAC staging was reported as clinical stage, utilizing pathologic stage only if the case did not undergo neoadjuvant therapy and the tumor was resected within 30 days after sample collection (Supplementary Tables S1 and S2).

Controls included two groups: (i) controls (healthy controls and individuals without pancreatic disease or symptoms, including hereditary high-risk individuals with normal surveillance imaging) and (ii) benign conditions (pancreatitis and IPMNs).

Healthy controls had no personal history of pancreatic disease or symptoms, no personal history of cancer (except BCC/SCC of skin), and no family history of pancreatic cancer. Healthy control participants were recruited from a neighboring geriatric primary care practice or via a University of Pittsburgh healthy-control recruitment application. If a participant had no prior pancreatic imaging, they were labeled as a healthy control based on the absence of symptoms/history suggestive of pancreatic disease.

Hereditary/familial controls included unaffected hereditary high-risk individuals undergoing surveillance for FPC- or a PDAC-associated germline mutation when imaging/EUS was normal, as well as hereditary/familial high-risk individuals (FPC- or PDAC-associated germline mutation) enrolled in pancreatic surveillance who had no clinical pancreatic disease but demonstrated benign parenchymal changes on imaging/EUS.

Benign conditions included chronic pancreatitis, recurrent acute pancreatitis, or acute pancreatitis at the time of biospecimen collection, with diagnoses labeled accordingly. Participants were recruited primarily from pancreas disease clinics. Benign conditions also contained the IPMN control group, which comprised clinically followed, stable pancreatic cysts consistent with IPMN without worrisome or high-risk features. Inclusion was restricted to cysts with a GNAS mutation detected by NGS to support the IPMN diagnosis. Participants were recruited through pancreas disease clinic visits or EUS, and this group also included high-risk surveillance individuals with small, stable, subcentimeter clinically diagnosed IPMN lesions.

Participants were excluded if they were previously analyzed by the group or had a comorbid or recent non-pancreatic malignancy, defined as active disease within 3 years prior to sampling, with the exception of basal cell carcinoma and squamous cell carcinoma of the skin.

### 2.2. COF Polymer-Based Enrichment of Plasma Proteins, Tryptic Digestion, and Proteomic Sample Preparation

Plasma samples, along with quality control samples consisting of pooled human plasma, were randomized into seven batches, each batch contained 96 samples. Enrichment and proteolytic digestion of plasma proteins were performed separately for each batch using magnetic-COF beads on AUTO-SP [34,35] as follows. For enrichment, 100 µg of magnetic-COF beads were used per sample. The beads were suspended by ultrasonication for 5 min and transferred into a 96-well PCR plate. The beads in each well were washed with 180 µL of washing solvent (0.1% formic acid [FA]) by pipette mixing.

After bead washing, 50 µL of 0.1% FA was added to each well, followed by aliquoting 125 µL of plasma from each sample into the corresponding wells and mixing. The mixture was incubated at room temperature for 30 min at 1600 rpm. Following incubation, the beads were washed with 180 µL of 0.1% FA for 1 min at 1600 rpm for a total of five cycles to remove high-abundance proteins.

On-bead proteolytic digestion was performed by adding 40 µL of 8 M urea lysis buffer containing 10 mM dithiothreitol and incubating at room temperature for 60 min at 1600 rpm. Samples were then alkylated with 15 mM iodoacetamide at room temperature for 30 min at 1600 rpm in the dark. Digestion was carried out by adding 1 mAU of Lys-C (Wako Chemicals, Richmond, VA, USA) and incubating at 37 °C for 60 min at 1600 rpm. The samples were subsequently diluted with 100 µL of 50 mM Tris-HCl (pH 8.0) and further digested with sequencing-grade modified trypsin (Promega, Madison, WI, USA) at 37 °C for 16 h at 1600 rpm.

Digested samples were acidified with 10 µL of 40% FA to a final pH < 3.0 and loaded onto EvoTips prior to LC–MS/MS analysis.

### 2.3. LC-MS/MS

LC–MS/MS analyses were performed on an Evosep One LC system (Evosep Biosystems, Odense, Denmark) coupled to a timsTOF HT mass spectrometer (Bruker Corporation, Billerica, MA, USA). As described previously [20], digested peptides were loaded onto EvoTips and desalted with 1% formic acid. Peptides were separated on a PepSep C18 analytical column (15 cm × 150 μm i.d., 1.5 μm; Bruker) positioned in a column oven (“column toaster”) maintained at 50 °C. Chromatographic separation was carried out using the Evosep 30 samples-per-day (30 SPD) preset gradient. Data were acquired in DIA-PASEF mode with a MS1 scan range of 100–1700 *m*/*z*, MS2 scan range of 338.6–1338.6 *m*/*z*, 1/K0 range of 0.70 to 1.45 V/s/cm^2^, and Ramp time of 85 ms.

### 2.4. Serum CA19-9 Measurement

Serum CA 19-9 was measured with the ST-AIA-PACK CA19-9 Immunoassay on the AIA-900-II immunoassay analyzer (Tosoh Bioscience, Grove City, OH, USA).

### 2.5. Bioinformatics

#### 2.5.1. MS Data Processing

The DIA raw files were searched against the UniProt/Swiss-Prot human protein sequence FASTA file using directDIA mode in the Spectronaut (Biognosys, Schlieren, Switzerland, version 19.9) [37]. The search setting was as follows. Mass tolerance of MS and MS/MS was set as dynamic with a correction factor of 1. Precursors were filtered by a Q value cutoff of 0.01. Carbamidomethyl (C) was set as a fixed modification. Acetyl (Protein N-term) and Oxidation (M) were set as variable modifications. The quantity of a protein was the average of the quantity of its top 3 peptides (stripped sequences). The data matrix was log_2_-transformed, median-normalized, and subjected to KNN-based imputation for proteins quantified in more than 40% of samples.

#### 2.5.2. Statistical Analysis

The unblinded dataset was used for model selection, hyperparameter tuning, final model derivation, and selection of clinical cutoff on model output index. Among models with comparable mean areas under the curve (AUC) from receiver-operating characteristic (ROC) curve analysis, the final model was selected to have the smallest standard deviation of AUC over cross-validation resamples for a greater model stability.

For the final model, a radial basis function (RBF) SVM was implemented using the caret package (version 7.0-1), with hyperparameters including the kernel width (σ) and regularization parameter (C) tuned via an explicit grid search. The σ grid was centered on a data-driven heuristic derived from pairwise distances between samples. Specifically, an initial estimate of σ was obtained using the sigest function (kernlab, version 0.9-33), applied to the centered and scaled data matrix, and the median estimate was used as the baseline (σ_0_). The tuning grid was defined as σ = σ_0_ × 2^−2,−1,0,1,2^. The cost parameter C was tuned over a logarithmic scale (2^−2^ to 2^8^) to balance the margin width against training errors. The final model used σ = 0.114 and C = 0.5. Model performance was assessed using repeated 10-fold cross-validation (20 repeats). Preprocessing (centering and scaling) was performed within each resampling iteration, with parameters estimated using only the training portion of each fold and then applied to the corresponding held-out samples to prevent information leakage.

The selected model was subsequently refit using all unblinded samples and a cutoff was determined to yield a 95% sensitivity on all PDAC cases in the unblinded samples.

The derived model with fixed cutoff was then applied to data of the blinded validation samples. The individual biomarkers ITIH3 and CEACAM1, and the multivariate model were compared to CA19-9 by ROC analysis, and specificity at a fixed 95% sensitivity. Four sets of comparisons were performed: (1) all PDAC cases vs. all controls; (2) normal controls vs. all PDAC cases; (3) benign controls vs. all PDAC cases; and (4) normal controls vs. stage I/II PDAC.

## 3. Results

### 3.1. Cohort Overview for Unblinded Training and Blinded Testing Sets

A total of 649 participants with matched serum and plasma samples from UPMC were analyzed. To develop the multivariate algorithm and pre-specify decision cutoffs, we randomly selected an unblinded training subset from the full cohort with stratification of clinical groups (Table 1, Supplementary Tables S1 and S2). The unblinded subset included 92 controls (50 healthy controls and 42 individuals with normal pancreas imaging), 84 PDAC cases, and 47 individuals with benign pancreatic conditions: 4 acute pancreatitis (AP), 34 chronic pancreatitis (CP), 7 acute recurrent pancreatitis, and 2 intraductal papillary mucinous neoplasms (IPMNs). The remaining 426 participants remained as an independent blinded validation set (167 controls, 166 PDAC cases, and 93 benign pancreatic conditions), which was only evaluated once by an honest broker who was not involved in model development.

### 3.2. Serum CA19-9 Analysis

From Table 1, among benign conditions, acute recurrent pancreatitis (13.3 U/mL), and acute pancreatitis (15.9 U/mL) showed similar median CA19-9 levels compared to the normal control groups. In contrast, CP (22.6 U/mL) and IPMN (24.7 U/mL) exhibited modestly higher CA19-9 levels than normal controls yet remained lower than PDAC. Together, these distributions highlight the limited specificity of CA19-9 in the setting of inflammatory or premalignant pancreatic disease and motivate the need for complementary markers to improve detection accuracy.

### 3.3. Application of MS-Based (COF) Polymer Enrichment to Plasm Proteome Analysis

Recently, polymer-based low-abundant blood protein enrichment strategies coupled with state-of-the-art MS workflows have expanded the number of detectable blood proteins and have been successfully applied to pancreatic cancer study [28,29,30]. Building on these advances, we applied them to plasma protein enrichment in the AUTO-SP workflow [34,35] followed by LC–MS/MS analysis on an Evosep-timsTOF-HT platform, which enabled quantification of more than 5000 plasma proteins, including many proteins previously implicated in pancreatic cancer in our prior studies [18,19,20]. Notably, ITIH3 and CEACAM1 candidates initially highlighted in our earlier serum glycoproteomics analyses [19], also demonstrated robust performance in COF-enriched plasma approach (Supplementary Table S3). We therefore evaluated whether incorporating ITIH3 and CEACAM1 with CA19-9 could improve diagnostic specificity while maintaining high sensitivity for PDAC detection.

### 3.4. Clinical Performance of CA19-9/ITIH3/CEACAM1 Marker Panel

**Figure 1:**
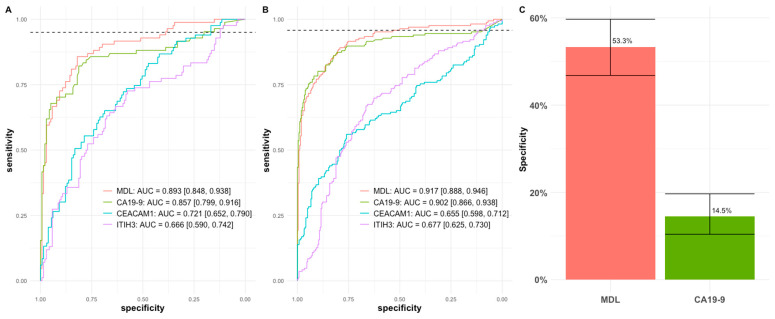
Comparison of multivariate model performance with CA19-9. (**A**) Comparison of ROC curves on the unblinded training samples used for model derivation. Horizontal dashed line indicates 95% sensitivity. A clinical threshold on the model output index was selected at 95% sensitivity. (**B**) Comparison of ROC curves on all blinded validation samples including healthy or normal controls, and patients with benign pancreatic conditions (see Tables). At the prefixed threshold, the model had a sensitivity of 95.7%, indicated by the horizontal dashed line. (**C**) At this prefixed cutoff, the model had a much-improved specificity at 53.3% (95% CI: 46.8–59.7%) over that of CA19-9 at 14.5% (95% CI: 10.4–19.7%) with statistical significance (*p* < 0.0001).

The performance of each individual marker and the three-marker multivariate model (MDL) was evaluated by receiver operating characteristic (ROC) analysis. Model training and cutoff selection were conducted using the unblinded training subset (Table 1 and Figure 1A), with healthy controls/normal pancreas participants and benign pancreatic conditions combined as the control group. In the training subset, CA19-9 was the best performing single marker (AUC 0.857; 95% CI: 0.799–0.916), while the three-marker MDL provided improved discrimination (AUC 0.893; 95% CI: 0.848–0.938) compared with CA19-9 alone. We then locked the model and prefixed a cutoff corresponding to 95% sensitivity (horizontal dashed line) to compare specificity at a clinically relevant operating point. When the MDL was applied to the independent blinded validation subset (Table 1 and Figure 1B), the MDL maintained a strong performance (AUC 0.917; 95% CI: 0.888–0.946), comparable to CA19-9 (AUC 0.902; 95% CI: 0.866–0.938) alone. At the prefixed cutoff of blinded validation (Figure 1C), the MDL achieved a sensitivity of 95.7% (Figure 1B, horizontal dashed line) and a higher specificity than CA19-9 alone (53.3% [95% CI: 46.8–59.7%] vs. 14.5% [95% CI: 10.4–19.7%]). Collectively, these results indicate that integrating plasma ITIH3 and CEACAM1 with serum CA19-9 can substantially improve specificity while preserving high sensitivity for PDAC detection.

**Figure 2:**
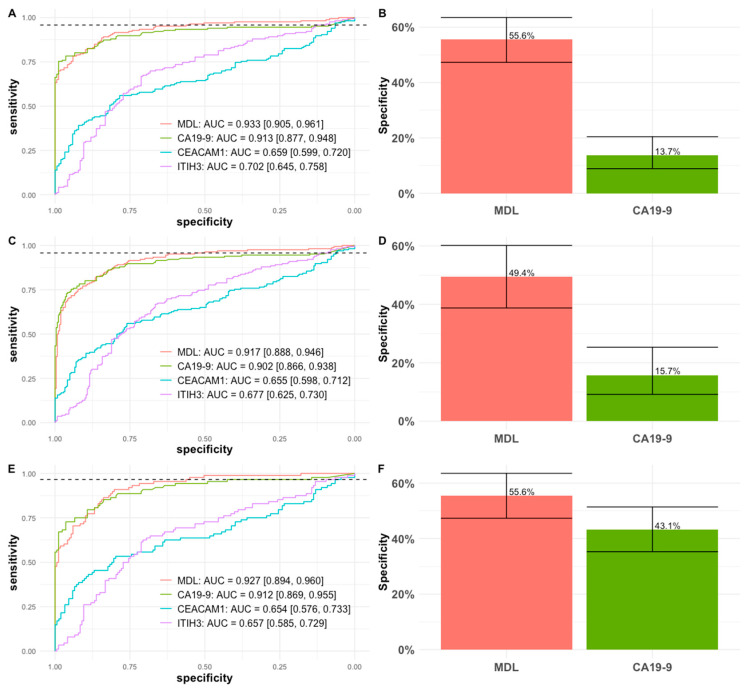
Comparison of the multivariate model with CA19-9 in blinded validation. (**A**) ROC curves between healthy control/normal pancreas and PDAC cases; (**B**) At the preset cutoff, MLD had a sensitivity of 95.7% in blinded validation, at the same sensitivity, the comparison of specificity between MDL and CA19-9 alone with healthy control/normal pancreas and PDAC cases; (**C**) ROC curves between the patients with benign pancreatic conditions and PDAC cases; (**D**) At the preset cutoff, MDL had a sensitivity of 95.7% in blinded validation, the comparison of specificity between MDL and CA19-9 alone with benign pancreatic conditions and PDAC cases. (**E**) ROC curves between healthy control/normal pancreas and stage 1/2 PDAC cases; (**F**) At the preset cutoff, MDL had a sensitivity of 96.6% to detect stage 1/2 PDAC in blinded validation, at the same sensitivity comparison of specificity between MDL and CA19-9 alone with healthy control/normal pancreas and stage 1/2 PDAC cases. The horizontal dashed line indicates the pre-fixed sensitivity threshold.

To further assess the robustness of our model within the blinded validation cohort, we performed analyses using alternative control group definitions (Figure 2A–D). When limiting controls to healthy controls plus normal pancreas participants (Table 1), the three-marker MDL showed better performance (AUC 0.933; 95% CI: 0.905–0.961; Figure 2A). At the prefixed cutoff, MDL had a sensitivity of 95.7%, delivering a substantially higher specificity than CA19-9 alone at the same sensitivity (55.6% vs. 13.7%; Figure 2B). We next evaluated a clinically challenging comparison by using benign pancreatic conditions as the only control group (Table 1). Although overall discrimination of MDL and CA19-9 was similar in this setting (AUC 0.917, 95% CI: 0.888–0.946 vs. AUC 0.902, 95% CI: 0.866–0.938; Figure 2C), the MDL maintained a higher specificity at the same fixed sensitivity (49.4% vs. 15.7%; Figure 2D), with the improvement remaining statistically significant at the prespecified cutoff. Finally, we evaluated the ability of the three-marker MDL to detect stage I and II PDACs from normal controls (Figure 2E,F). The MDL similarly had improved specificity at the high sensitivity range of the ROC curve. At the prefixed cutoff, the MDL had a sensitivity of 96.6% (95% CI: 89.5–99.1%) and a specificity of 55.6%. At the same sensitivity, CA19-9 had a specificity of 43.1%. The improvement in specificity over CA19-9 in the above comparisons were all statistically significant. For Figure 2F, even though the 95% CIs overlap, the difference was still statistically significant in a paired test (*p* = 0.0015, McNemar’s chi-squared). Overall, these analyses demonstrate that the specificity greatly improved by integrating ITIH3 and CEACAM1 with CA19-9 and was robust to the control–group composition and early-stage PDACs.

Finally, the blinded validation samples included 77 non-PDAC controls that had familiar pancreatic cancer history or PDAC-associated mutations (FPC/Mut). The performance of the model-using controls with and without FPC/Mut were comparable: ROC AUCs were 0.910 95% CI [0.878, 0.941] vs. 0.935 95% CI [0.905, 0.965] with controls without and with FPC/Mut, respectively; at the cutoff preset in training and a corresponding sensitivity of 95.7%, the specificities were 51.1% 95% CI [45.5%, 58.8%] and 58.6% 95% CI [46.2%, 70.0%] with controls without and with FPC/Mut, respectively.

## 4. Discussion

Pancreatic cancer remains a highly lethal malignancy largely because most patients present with advanced-stage disease. Blood-based testing offers the potential for a minimally invasive strategy for detection and monitoring disease. However, the current clinical standard, serum CA19-9, has limited specificity, particularly at high-sensitivity operating points, leading to the possibility of marked non-malignancy cases as cancer [16,23]. To address this limitation, we applied our in-house synthetic COF polymers to enrich plasma proteins from 649 participants and performed deep profiling by LC–MS/MS, while measuring CA19-9 in matched serum by an established immunoassay to enable integrated multi-marker modeling (Table 1). Using an unblinded subset for model training and a prefixed cutoff followed by independent blinded validation, we confirmed two robust PDAC-associated candidate plasma proteins, ITIH3 and CEACAM1, consistent with our prior reports [19,20]. Incorporating ITIH3 and CEACAM1 with CA19-9 in a three-marker multivariate model substantially improved specificity in the clinically relevant high-sensitivity range (Figure 1). In blinded validation, at the prespecified cutoff from training, it had a sensitivity of 95.7% and a specificity of 53.3%, a significant improvement both statistically (*p* < 0.0001, McNemar’s test) and clinically over that of 14.5% for CA19-9 alone, supporting this panel as a promising approach to improve specificity while preserving high sensitivity for PDAC detection (Figure 1C). Moreover, we performed sensitivity analyses using different control group definitions to evaluate performance across distinct clinical populations. When controls were restricted to healthy controls and participants with normal pancreas findings, the three-marker panel achieved its highest performance at an AUC of 0.933 (Figure 2A). In contrast, when the control group was limited to benign pancreatic conditions, excluding healthy controls and normal pancreas participants, reduced the overall performance (Figure 2C), reflecting the biological and clinical similarity between benign pancreatic disease and PDAC. Importantly, across both comparisons, the three-marker panel consistently delivered substantially higher specificity than CA19-9 alone at the prespecified high-sensitivity operating point (Figure 2B,D).

Polymer-based enrichment coupled with LC–MS/MS enables deep and comprehensive profiling of the blood proteome, creating an opportunity to systematically investigate disease-associated protein changes that may be observed in circulation, an especially important advantage for largely asymptomatic, early-stage invasive malignancies such as pancreatic cancer. In the present study, we focused on a targeted evaluation of PDAC-associated blood proteins previously identified in our prior proteomics and glycoproteomics studies. While this strategy facilitated rapid development and validation of a clinically actionable three-marker panel, it does not fully leverage the depth of the more than 5000 quantified plasma proteins generated by the COF-enrichment workflow. In future work, we will expand discovery and validation to additional candidates from this dataset, incorporate network-level prioritization, and assess whether larger multi-marker panels or alternative models further improve performance, particularly for early-stage disease and in clinically challenging benign conditions.

Several limitations of this study should be acknowledged. CA19-9 at its typical clinical cutoff can be elevated in a range of benign gastrointestinal conditions, inflammatory states, and non-pancreatic neoplasms, which potentially limit cancer specificity [38]. To overcome this limitation, we incorporated two additional PDAC-associated blood biomarkers, ITIH3 and CEACAM1, with distinct biological functions. ITIH3 is a hyaluronic-acid-binding protein involved in extracellular matrix regulation [39], and CEACAM1 is an immunoglobulin superfamily adhesion molecule implicated in cell–cell interactions and signaling [40]. While both ITIH3 and CEACAM1 have been reported as blood-based biomarkers for pancreatic cancer [41,42,43], they have also been associated with other cancer types [44,45,46], suggesting that the proposed panel may improve discrimination within pancreatic disease controls yet still may not be strictly pancreatic cancer-specific. It is likely therefore that CEACAM1 and ITIH3 are not specific to PDAC. However, when they are combined with serum CA19-9 in a multivariate algorithm, the expectation is that at the same high-sensitivity level, the additional PDAC cases captured by the two markers will allow CA19-9 to operate at a higher effective cutoff (figuratively) to yield an overall better specificity for PDAC. Furthermore, the three-marker model is configured for ruling out low-risk subjects in populations with elevated risk of PDAC. A better specificity at high sensitivity which improves negative predictive value clinically is more important than being specific to pancreatic cancer. Moreover, the Lewis antigen-negative population warrants further investigation in future studies with a specifically designed cohort. Finally, the current study is based on clinical specimens from a single site with a case–control design. The estimated performance, even in blinded validation, could be affected by site-specific factors and biases by the proportion of samples of different clinical groups in controls and cases. Further studies with large sample sizes, and preferably multi-site cohort collection are needed to further validate the findings.

## 5. Conclusions

In summary, using a large and matched plasma–serum cohort with prespecified subsets for modeling and blinded independent validation, we show that integrating two COF-enriched plasma proteins (ITIH3 and CEACAM1) with serum CA19-9 yields higher specificity for PDAC detection compared to CA19-9 alone. Across validation analyses with varying clinical groups for control and cases, the three-marker model consistently preserved high sensitivity while increasing specificity, thereby reducing the false-positive burden that limits CA19-9-based detection of PDAC which has a low-prevalence. These results highlight the value of deep plasma proteome profiling for identifying complementary circulating markers and provide a strong rationale for prospective, multicenter studies to determine the clinical utility of the CA19-9/ITIH3/CEACAM1 panel, including performance in high-risk populations and earlier-stage PDAC.

## Figures and Tables

**Table 1:** Study population of the 649 participants.

Clinical Group	Diagnosis	CA19-9 (U/mL)		Blinded		Total
		Mean, 95% CI *	Median	No (Training)	Yes (Validation)	
Controls	healthy control	19.86 [16.88; 22.84]	15.0	50 (35.7%)	90 (64.3%)	140 (21.6%)
	hereditary/familial **	16.74 [14.18; 19.29]	12.5	42 (35.3%)	77 (64.7%)	119 (18.3%)
Benign Conditions	acute pancreatitis	54.54 [−0.95; 110.03]	15.9	4 (36.4%)	7 (63.6%)	11 (1.7%)
	chronic pancreatitis	56.38 [12.44; 100.32]	22.6	34 (40.5%)	50 (59.5%)	84 (12.9%)
	acute recurrent pancreatitis	18.83 [12.35; 25.31]	13.3	7 (28.0%)	18 (72.0%)	25 (3.9%)
	IPMN ***	29.12 [19.79; 38.44]	24.7	2 (10.0%)	18 (90.0%)	20 (3.1%)
PDAC	Resected Stage 1	436.38 [63.33; 809.44]	91.3	17 (25.8%)	49 (74.2%)	66 (10.2%)
	Resected Stage 2	288.24 [190.23; 386.25]	104.2	27 (40.9%)	39 (59.1%)	66 (10.2%)
	Resected Stage 3	394.48 [211.78; 577.18]	273.0	1 (7.1%)	13 (92.9%)	14 (2.2%)
	Unresected Met/Late Stage	5469.04 [5.10 × 10^1^; 1.09 × 10^4^]	547.2	39 (37.5%)	65 (62.5%)	104 (16.0%)
All		975.8, [97.0; 1854.6]	27.7	223 (34.4%)	426 (65.6%)	649 (100.0%)

* Bootstrap estimated. ** Include low-risk (2 patients with subcentimeter pancreatic cyst) and 55 patients with benign parenchymal findings. *** On cyst fluid analysis all had a GNAS mutation and no worrisome or high-risk features by imaging or genomic markers so classified as IPMN with low-grade dysplasia.

## Data Availability

The data presented in this study are available on request from the corresponding authors.

## Supplementary Materials

The following supporting information can be downloaded at: https://www.mdpi.com/article/10.3390/cancers18091397/s1, Table S1: Detailed information of study population; Table S2: Key baseline characteristics by study group; Table S3: Expression of proteins in the biomarker panel.

## Abbreviations

The following abbreviations are used in this manuscript:

PDAC	pancreatic ductal adenocarcinoma
CA19-9	carbohydrate antigen 19-9
MS	mass spectrometry
COF	covalent organic framework
AUTO-SP	automated sample preparation
UPMC	University of Pittsburgh Medical Center
PCDC	pancreatic cancer detection consortium
EDRN	early detection research network
FA	formic acid
SVM	support vector machine
AUC	area under the curve
ROC	receiver operating characteristic
SD	standard deviation
AP	acute pancreatitis
CP	chronic pancreatitis
IPMN	intraductal papillary mucinous neoplasm
MDL	multivariate model
FPC/Mut	familial pancreatic cancer history or PDAC-associated mutations
